# Predictive Clinical Factors for Endoscopic Ultrasound Diagnosis in Undiagnosed Common Bile Duct Dilatation on Cross-Sectional Imaging

**DOI:** 10.7759/cureus.105841

**Published:** 2026-03-25

**Authors:** Mayank Chotrani, Jay Chudasama, Sanjay Chandnani, Sameet T Patel, Neil Prabhu, Yogita Kokate, Harsh Gandhi, Rishikesh Malokar, Mavuri Vishal, Deepika Pandey, Shubham Jain, Suwayed Khan, Pravin M Rathi

**Affiliations:** 1 Gastroenterology, Topiwala National Medical College and Bai Yamunabai Laxman (BYL) Nair Charitable Hospital, Mumbai, IND; 2 Gastroenterology, Lokmanya Tilak Municipal Medical College and Lokmanya Tilak Municipal General Hospital, Mumbai, IND

**Keywords:** ct, dilated cbd, eus, inconclusive cross-sectional imaging, magnetic resonance cholangiopancreatography, mrcp

## Abstract

Introduction

Dilatation of the common bile duct (CBD) without an identifiable etiology on computed tomography (CT) or magnetic resonance cholangiopancreatography (MRCP) is a common clinical challenge and may lead to repeated investigations and unnecessary invasive procedures. Endoscopic ultrasound (EUS), due to its close proximity to the biliary tree, has emerged as a promising diagnostic tool. This study aimed to evaluate the diagnostic yield of EUS in patients with unexplained CBD dilatation on CT/MRCP and identify clinical predictors of positive EUS findings.

Methods

This was a prospective observational study conducted at a tertiary care center in Western India from January 2023 to January 2024. Patients with a dilated CBD (>6 mm with gallbladder in situ or >8 mm post-cholecystectomy) and no identifiable cause on CT or MRCP were included. EUS was performed within two weeks of cross-sectional imaging, and findings were confirmed with ERCP or biopsy where indicated. Clinical, laboratory, and imaging variables were analysed to identify predictors of positive EUS diagnosis.

Results

Out of 525 patients who underwent EUS, 75 met the inclusion criteria. The mean age was 50.6 ± 15.9 years, and 56% were female. A definitive diagnosis was established in 41 patients (54.7%), with choledocholithiasis (33.3%) and benign biliary strictures (10.7%) being the most common etiologies. Multivariate analysis identified age ≥49.5 years, alkaline phosphatase ≥181 IU/L, and CBD diameter ≥8.75 mm on cross-sectional imaging as independent predictors of a positive EUS diagnosis. The combined area under the receiver-operating characteristic curve (AUROC) for these variables was 0.746.

Conclusion

EUS established a definitive diagnosis in approximately half of patients with unexplained CBD dilatation. Its ability to detect small stones makes it a valuable diagnostic modality. Age, elevated alkaline phosphatase, and increased CBD diameter were significant predictors of diagnostic yield, supporting the selective use of EUS in this population.

## Introduction

Dilated common bile duct (CBD) without any identifiable etiology on a computed tomography (CT) or magnetic resonance cholangiopancreatography (MRCP) is a common finding with ever-increasing use of radiological investigations in the current clinical scenario. Undiagnosed dilated CBD can lead to patient distress and anxiety. It can also lead to a burden on the healthcare provider due to a lack of official guidelines for the approach to an undiagnosed dilated CBD on cross-sectional imaging, leading to repeated patient visits to the outpatient department and difficulty in arriving at a decisive diagnosis and management plan.

Endoscopic ultrasound (EUS) can help in imaging the CBD from greater proximity and hence be a useful tool in the armamentarium for the evaluation of patients with an undiagnosed dilated CBD. EUS has shown encouraging results in previously conducted studies for providing etiology in undiagnosed dilated CBD [1-3]. However, opting for an interventional procedure like EUS is frequently questioned and typically demands thorough justification.

There is limited data from India with regard to this. Thus, the objective of this study was to evaluate the diagnostic yield of EUS in patients with unexplained dilated CBD on CT/MRCP and to identify clinical, biochemical, and imaging predictors associated with positive EUS findings.

## Materials and methods

This was a prospective observational study conducted in Topiwala National Medical College and BYL Nair Charitable Hospital, Mumbai, Maharashtra, India, from January 2023 to January 2024. The study was reviewed and approved by the Ethics Committee for Academic Research Projects (ECARP), PG Academic Committee, Topiwala National Medical College and BYL Nair Charitable Hospital (approval number: ECARP/2022/64).

Participants

Inclusion criteria were: Adult patients with a CBD diameter of > 6 mm with gallbladder in situ or > 8 mm in patients who have undergone a cholecystectomy on CT/MRCP without any identifiable cause. Exclusion criteria included: Patients with (i) an identifiable cause of dilated CBD on CT/MRCP, (ii) a history of previous ERCP, (iii) a history of pancreaticobiliary surgery leading to altered anatomy, (iv) pregnancy, and (v) patients unfit for sedation required for EUS examination.

EUS was performed on 525 patients during the study period. Of 525 patients, 75 patients fulfilled the eligibility criteria and were included.

Procedure details

EUS examination was performed using a longitudinal convex-array echoendoscope (EG38-J10UT; Pentax Corporation, Tokyo, Japan) in left lateral position. Procedures were conducted under intravenous sedation with propofol, administered by a trained anesthesiologist. EUS was performed by an experienced endo-sonographer who had performed >1000 EUS procedures. EUS examinations were conducted at one to three weeks following symptom onset and within two weeks of the cross-sectional imaging study. The diameter of the CBD was measured at its maximum width on cross-sectional imaging or EUS.

The EUS findings were confirmed with either ERCP with stone removal in case of choledocholithiasis or histological confirmation after fine needle biopsy in case of malignancy. Rosemont criteria [4] were used for diagnosing chronic pancreatitis. The diagnosis of benign biliary stricture was established by the lack of disease progression on follow-up MRCP at six months.

Statistics

Quantitative variables were expressed as mean ± standard deviation (SD) or median (interquartile range (IQR)), depending on the normality of distribution. The Kolmogorov-Smirnov test was used to assess the normality of the distribution. Continuous variables were compared using Student’s t-test for normally distributed data, and the Mann-Whitney U test for non-normally distributed data. Categorical variables were compared using the Chi-squared test.

Univariate and multivariate logistic regression analyses were performed to calculate odds ratios (OR) and 95% confidence intervals (CI) for predictors of the outcome. Variables with a p-value < 0.05 in univariate analysis were included in the multivariate model. A p-value < 0.05 was considered statistically significant. All statistical analyses were performed using IBM SPSS Statistics for Windows, version 28 (IBM Corp., Armonk, Washington, United States).

## Results

A total of 75 patients were included in the study, who had dilated CBD without any etiology on MRCP or CT. MRCP was done in 38 patients and CT in 27 patients; 10 underwent both CT and MRCP. CT scans were performed using 16-slice or higher scanners, and MRI was conducted with machines of 1.5 Tesla or higher field strength.

Baseline characteristics

Among the 75 patients, the majority were female (56 %). The mean age was 50.6 ± 15.9 years. Abdominal pain was the most common symptom present in 70 (93.3 %) patients, followed by loss of appetite in 19 (25.3 %). Demographic and baseline profile of study participants are shown in Table 1.

**Table 1:** Demographic and baseline profile of study participants (N=75)

Variable	Value
Number of patients, n	75
Female, n (%)	42 (56%)
Age (years), mean ± SD	50.6 ± 15.9
Clinical Presentation, n (%)	
Abdominal Pain	70 (93.3 %)
Fever	7 (9.3 %)
Jaundice	14 (18.7 %)
Abdominal Distension	4 (5.3 %)
Loss of appetite	19 (25.3 %)
Significant loss of weight	17 (22.7 %)
Past History, n (%)	
Past history of pancreatitis	2 (2.7 %)
Past history of biliary colic	8 (10.7 %)
History of cholecystectomy	4 (5.3 %)
Past history of jaundice	4 (5.3 %)
Presence of comorbidity	7 (9.3%)
Personal History; n (%)	
History of significant alcohol intake	2 (2.7 %)
History of smoking	9 (12 %)
Laboratory parameters	
Hemoglobin (g/dl), mean ± SD	11.3 ± 1.85
White Blood Cell Count (/mm^3^), median (IQR)	6900 (2700, 23000)
Platelet Count (/mm^3^), median (IQR)	236000 (82000, 514000)
Aspartate Aminotransferase (IU/L), median (IQR)	37 (12, 565)
Alanine Aminotransferase (IU/L), median (IQR)	35 (7, 640)
Total Bilirubin (mg/dl), median (IQR)	0.9 (0.3, 16)
Alkaline Phosphatase (IU/L), median (IQR)	255 (63, 918)
CBD size (in mm), mean ± SD	
Cross sectional imaging (CT/MRCP)	9.95 ± 2.45
EUS	9.42 ± 2.74
Outcomes on EUS, n (%)	
Choledocholithiasis	25 (33.3 %)
Benign biliary stricture	8 (10.7 %)
Chronic Pancreatitis	3 (4 %)
Distal CBD mass	2 (2.7 %)
Ampullary mass	2 (2.7 %)
Periampullary diverticulum	1 (1.3 %)
Dilated CBD without any identifiable etiology	25 (33.3 %)
Normal CBD	9 (12 %)

EUS findings

Mean CBD diameter on EUS was 9.42 ± 2.74 mm. Among all the patients who underwent EUS, 45.3% were undiagnosed (dilated CBD without any etiology and normal-sized CBD). Amongst those with an etiology, the most common pathology was choledocholithiasis seen in 25 (33.3%) patients, followed by benign biliary stricture seen in eight (10.7 %) patients (Table 1).

Comparison of variables between patients with positive and negative EUS results

Among the different demographic and baseline clinical, laboratory, and EUS variables, age and clinical jaundice at baseline were significantly higher in the group with a positive diagnosis on EUS. Median value of total bilirubin, alkaline phosphatase, and mean value of CBD diameter on cross-sectional imaging and EUS were significantly higher in the group with a positive diagnosis on EUS (Table 2).

**Table 2:** Comparison of clinical, laboratory, and imaging variables between patients with and without an established EUS diagnosis Continuous variables were analyzed using Student’s t-test or Mann–Whitney U test based on distribution. Categorical variables were compared using the Chi-square test or Fisher’s exact test. Corresponding test statistics are reported alongside p-values. A p-value <0.05 was considered statistically significant. CBD: common bile duct; EUS: endoscopic ultrasound

Variables	EUS Diagnosis			
	No (n=36)	Yes (n=39)	Test statistic	P value
Age (years), median (IQR)	54 (26, 80)	46.5 (20, 76)	U = 532.5	0.043
Male, n (%)	12 (33.33%)	21 (53.8 %)	χ² = 2.42	0.074
Abdominal Pain, n (%)	34 (94.4 %)	36 (92.3 %)	-	0.711
Fever, n (%)	3 (8.3 %)	4 (10.2%)	-	0.775
Jaundice, n (%)	3 (8.3 %)	11 (28.2 %)	χ² = 3.65	0.027
Loss of appetite, n (%)	9 (25%)	10 (25.6%)	χ² = 0.00	0.949
Loss of weight, n (%)	9 (25%)	8 (20.5%)	χ² = 0.04	0.643
Past history of Pancreatitis, n (%)	1 (2.7 %)	1 (2.5 %)	-	0.954
Past history of biliary colic, n (%)	6 (16.6 %)	2 (5.1 %)	-	0.106
Hemoglobin, mean ± SD	11.24 ± 1.44	11.36 ± 2.18	t = −0.28	0.787
WBC, median (IQR)	6630 (3700, 17900)	6900 (2700, 23000)	U = 534.0	0.147
Platelet, median (IQR)	216500 (126000, 433000)	242000 (82000, 514000)	U = 686.5	0.926
Total Bilirubin, median (IQR)	0.65 (0.3, 7)	1 (0.3, 16.2)	U = 529.0	0.022
Aspartate Aminotransferase, median (IQR)	36 (12, 372)	29.5 (10, 542)	U = 638.0	0.169
Alanine Aminotransferase, median (IQR)	29 (10, 548)	39 (7, 640)	U = 633.5	0.397
Alkaline Phosphatase, median (IQR)	241 (63, 498)	336 (79, 918)	U = 470.5	0.002
CBD size on CT/MRI, mean ± SD	9.053 ± 1.53	10.79 ± 2.84	t = −3.33	0.002
Gall bladder calculi, n (%)	22 (61.1 %)	15 (38.5 %)	χ² = 2.99	0.054

Variables associated with positive outcomes on EUS

Age, jaundice, total bilirubin, alkaline phosphatase, and cross-sectional CBD diameter were identified as predictors of EUS diagnosis in univariate analysis. Multivariate regression analysis identified age, alkaline phosphatase levels, and CBD diameter on cross-sectional imaging as independent predictors of a positive EUS diagnosis (Table 3).

**Table 3:** Univariate and multivariate logistic regression analysis of demographic, clinical, and EUS parameters - Not included in multivariate analysis CBD: common bile duct; EUS: endoscopic ultrasound

Parameter	Univariate P-value	Univariate OR (95 % CI)	Multivariate P-value	Multivariate OR (95% CI)
Age	0.043	1.032 (1.001 – 1.064)	0.049	0.962 (0.926 – 1.000)
Abdominal Pain	0.657	1.500 (0.111 – 4.48)	-	-
Fever	0.775	1.257 (0.261 – 6.047)	-	-
Jaundice	0.037	4.321 (1.096 – 17.046)	0.424	3.621 (0.154 – 84.912)
Abdominal Distension	0.364	2.917 (0.289 – 29.398)	-	-
Loss of appetite	0.949	1.034 (0.365 – 2.933)	-	-
Loss of weight	0.643	0.774 (0.262 – 2.287)	-	-
Past H/o pancreatitis	0.954	0.921 (0.055 – 15.292)	-	-
Past h/o biliary colic	0.125	0.270 (0.051 – 1.437)	-	-
H/o Cholecystectomy	0.364	2.917 (0.289 – 29.398)	-	-
Comorbidity	0.095	6.364 (0.727 – 55.721)	-	-
Hemoglobin	0.783	1.035 (0.809 – 1.324)	-	-
WBC	0.158	1.000 (1.000 – 1.000)	-	-
Platelet	0.924	1.000 (1.000 – 1.000)	-	-
Total Bilirubin	0.046	1.304 (1.005 – 1.692)	0.124	0.688 (0.426 – 1.109)
Aspartate Aminotransferase	0.194	1.004 (0.998 – 1.010)	-	-
Alanine Aminotransferase	0.401	1.002 (0.997 – 1.006)	-	-
Alkaline Phosphatase	0.007	1.005 (1.001 – 1.008)	0.019	0.996 (0.992 – 0.999)
Cross-sectional CBD	0.006	1.535 (1.133 – 2.079)	0.036	0.712 (0.518 – 0.978)
Gall bladder calculi	0.052	0.398 (0.157 – 1.714)	-	-

**Figure 1:**
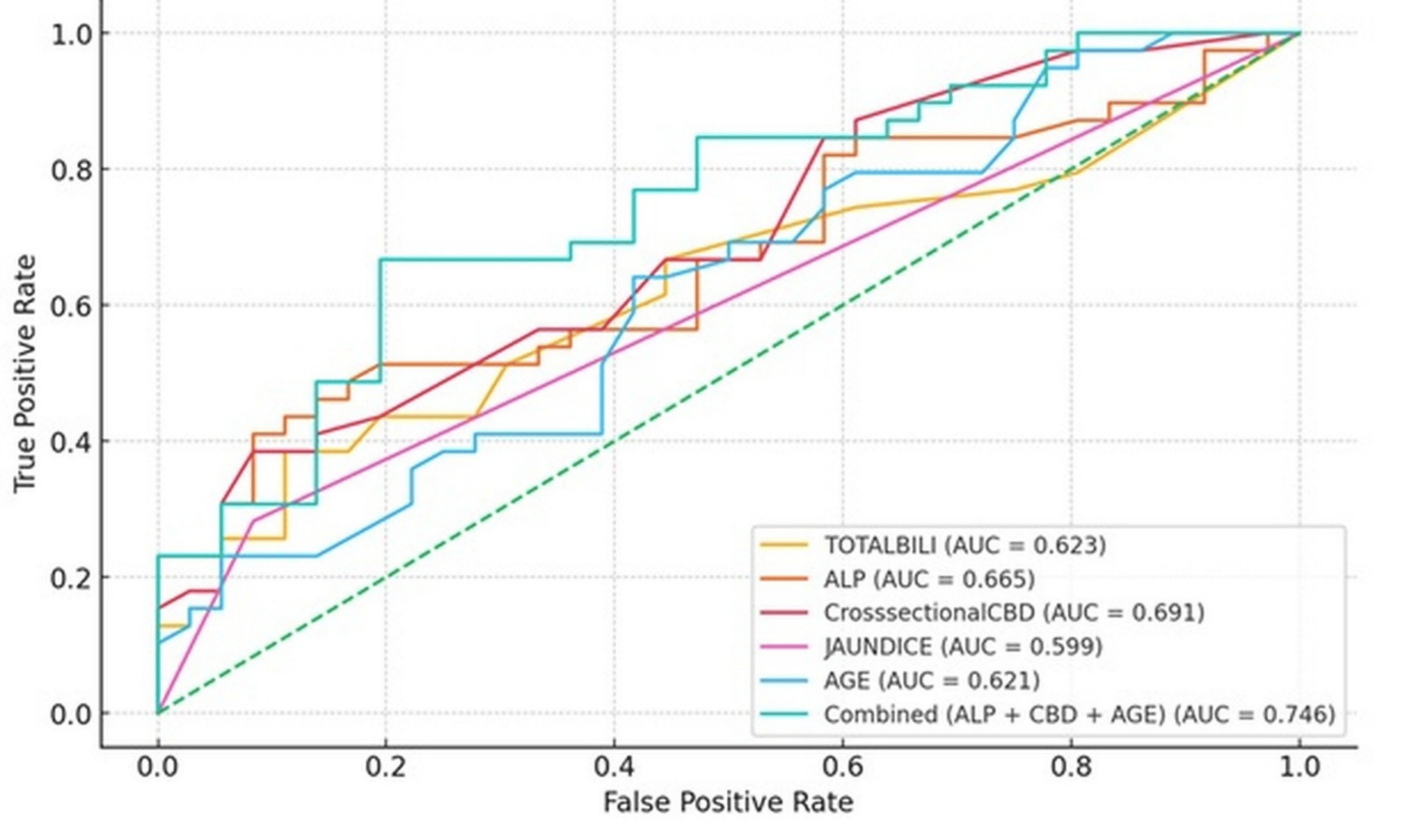
ROC Curves for individual predictors and combined model ROC curves showing the diagnostic performance of individual predictors—total bilirubin (AUC = 0.623), ALP (AUC = 0.665), CBD diameter on cross-sectional imaging (AUC = 0.691), clinical jaundice (AUC = 0.599), and age (AUC = 0.621)—and the combined model (ALP + CBD diameter + age; AUC = 0.746); the diagonal dashed line represents no discrimination (AUC = 0.50). ROC curves evaluate diagnostic accuracy; higher AUC values indicate better discrimination, with the combined model outperforming individual predictors. ALP: alkaline phosphatase; TOTALBILI: total bilirubin; CBD: common bile duct; AUC: area under the curve; ROC: receiver operating characteristic

A combined area under the receiver-operating characteristic curve (AUROC) of 0.746 was observed when age, alkaline phosphatase levels, and CBD diameter on cross-sectional imaging were analyzed together (Figure 1).

The optimal cutoff values predictive of a positive EUS diagnosis were 49.5 years for age, 181 IU/L for alkaline phosphatase, and 8.75 mm for CBD diameter.

## Discussion

Dilated CBD of indeterminate etiology is frequently observed on cross-sectional imaging. The sensitivity and specificity of MRCP for detecting choledocholithiasis are 92% and 97%, respectively, while for biliary malignancies, they are 88% and 95% [5]. In comparison, the sensitivity of CT for detecting CBD stones ranges from 69% to 88% [6]. For biliary malignancies, CT demonstrates a sensitivity and specificity ranging from 84% to 90% [7].

The limitations of MRCP in the evaluation of biliary tree pathologies include issues with visualizing small stones (less than 5 mm), difficulty in differentiating between benign and malignant strictures, potential for misinterpreting artifacts or normal variants as pathology, and the ampulla of Vater sometimes becomes a blind spot, leading to failure of detection of ampullary neoplasms [8-10]. CT also has similar issues with additional problems of radiation exposure and inferior characterization of choledocholithiasis.

EUS, with its anatomical proximity to the pancreaticobiliary system, serves as a valuable diagnostic modality in assessing unexplained CBD dilatation. EUS, apart from being a diagnostic tool in finding a cause for dilated CBD, also provides an opportunity in some of these cases to biopsy mass lesions to provide a histological diagnosis. EUS has advantages as it can detect smaller stones (< 5 mm). [11] EUS facilitates accurate assessment of ampullary neoplasms, given its spatial closeness to the duodenum and the ability to perform tissue sampling concurrently [10,12].

Several prospective as well as retrospective studies in the past have assessed the efficiency of EUS in patients with undiagnosed dilated CBD noted on CT or MRI [2,13]. Mahajan et al. reported a diagnostic yield of approximately 56% [1], and Rana et al. reported a yield of 50% [3], both of which are comparable to the 54.7% yield observed in our study.

In the present study, 34 patients (45.3%) remained undiagnosed even after EUS, including nine with normal CBD size and 25 with dilated CBD without an identifiable cause. Previously conducted studies showed similar results, where the majority of patients were undiagnosed after EUS [1,3]. In our study, some of the patients with unexplained CBD dilatation (n=25) may have had spontaneously passed stones, with EUS performed before the CBD had returned to normal size. This highlights a potential advantage of EUS, as it may help avoid unnecessary invasive procedures like ERCP. In a retrospective study by Quispel et al., 169 (63%) patients were able to avoid ERCP following EUS evaluation [14]. Similar benefits have been demonstrated in other studies, where EUS-guided ERCP has been associated with reduced complication rates related to ERCP [15-17].

The most common pathologic etiology in our study was choledocholithiasis, present in 25 (33.3 %) patients, followed by benign biliary stricture seen in eight (10.7%) patients. A similar etiological profile was reported in a study by Rana et al., wherein choledocholithiasis was the most common etiology [3]. In a study by Sotoudehmanesh et al., the most common pathology seen was CBD calculi (21.1%) [18].

On multivariate logistic regression analysis, age, alkaline phosphatase, and CBD diameter on cross-sectional imaging were found to be significant. Some studies have given prediction models for selecting the right patients for EUS examination and reducing unnecessary EUS examination, but have not been validated in larger cohorts. In a retrospective study by Raza et al., a predictive model incorporating age, history of ERCP, abdominal pain, and CBD diameter demonstrated a sensitivity of 92-100% and a specificity of 91-93% for diagnosing etiologies on EUS [19]. Similarly, in a study by Mahajan et al., serum bilirubin, CA 19-9, and CBD diameter were predictive of a positive diagnosis on EUS [1].

Strengths and limitations

This study has several strengths. First, its prospective design allowed for systematic and standardized data collection, reducing the risk of recall and selection bias. The inclusion of patients based on strict criteria, CBD dilatation without identifiable etiology on CT or MRCP, ensured a homogenous study population and enhanced internal validity. EUS was performed within two weeks of cross-sectional imaging, minimizing diagnostic delays and potential changes in disease status. Additionally, all procedures were conducted by a highly experienced endo-sonographer, which reduces variability in interpretation and increases the reliability of findings. Finally, the use of multivariate logistic regression and AUROC analysis enabled the identification and validation of independent predictors of a positive EUS diagnosis.

However, the study has certain limitations. It was conducted at a single tertiary care center, which may limit generalizability to broader clinical settings. Despite a large initial cohort, the small sample size met the inclusion criteria, potentially reducing statistical power. Lastly, the use of both CT and MRCP (with some patients undergoing both) introduced heterogeneity in baseline imaging, which may have influenced the diagnostic outcomes.

## Conclusions

In patients with a dilated CBD and no identifiable etiology on cross-sectional imaging, EUS provides meaningful additional diagnostic yield. Nearly half of such patients have an underlying cause that can be detected on EUS, most commonly biliary stone disease and benign strictures. The likelihood of identifying a causative pathology is higher in older patients and in those with clinical jaundice, cholestatic biochemical abnormalities, and greater bile duct dilatation on prior imaging. A combination of readily available clinical, laboratory, and imaging parameters can help stratify patients who are most likely to benefit from EUS, supporting its targeted use as a complementary investigation when conventional imaging is inconclusive.
